# Tropical larval and juvenile fish critical swimming speed (U-crit) and morphology data

**DOI:** 10.1038/s41597-022-01146-3

**Published:** 2022-02-10

**Authors:** Rebecca Fisher, Jeffrey M. Leis, J. Derek Hogan, David R. Bellwood, Shaun K. Wilson, Suresh D. Job

**Affiliations:** 1 Australian Institute of Marine Science Crawley, Crawley, Western Australia Australia; 2 University of Western Australia Oceans Institute and School of Plant Biology, Crawley, Western Australia Australia; 3 Institute for Marine and Antarctic Studies, University of Tasmania, Hobart, Tasmania 7001 Australia; 4 Department of Ichthyology, Australian Museum Research Institution, William Street, Sydney, New South Wales 2001 Australia; 5 Department of Life Sciences Texas A&M University – Corpus Christi 6300 Ocean Dr., Unit 5892, Corpus Christi, Texas 78412 USA; 6 Department of Marine Biology, James Cook University, Townsville, Queensland 4811 Australia; 7 Marine Science Program, Science and Conservation Division, Department of Biodiversity, Conservation and Attractions, Kensington, Western Australia 6151 Australia; 8 Batavia Coast Maritime Institute, Central Regional TAFE, Geraldton, Western Australia 6530 Australia

**Keywords:** Developmental biology, Behavioural ecology, Physiology, Animal behaviour, Animal physiology

## Abstract

Fish swimming capacity is a key life history trait critical to many aspects of their ecology. U-crit (critical) swimming speeds provide a robust, repeatable relative measure of swimming speed that can serve as a useful surrogate for other measures of swimming performance. Here we collate and make available one the most comprehensive datasets on U-crit swimming abilities of tropical marine fish larvae and pelagic juveniles, most of which are reef associated as adults. The dataset includes U-crit speed measurements for settlement stage fishes across a large range of species and families obtained mostly from field specimens collected in light traps and crest nets; and the development of swimming abilities throughout ontogeny for a range of species using reared larvae. In nearly all instances, the size of the individual was available, and in many cases, data include other morphological measurements (e.g. “propulsive area”) useful for predicting swimming capacity. We hope these data prove useful for further studies of larval swimming performance and other broader syntheses.

## Abstract (summary)

**Table:** 

Measurement(s)	Fish swimming speed • Fish morphology
Technology Type(s)	Swimming flume • Image analysis
Factor Type(s)	location • region • date
Sample Characteristic - Organism	Actinopteri
Sample Characteristic - Environment	marine water body
Sample Characteristic - Location	Great Barrier Reef • Caribbean Region • Morea • Taiwan Island

Machine-accessible metadata file describing the reported data: 10.6084/m9.figshare.16988074

## Background & Summary

Most tropical teleost fishes that are demersal as adults have a pelagic larval phase, in which eggs and very young larvae are swept away from spawning sites by ocean currents into open waters, where they develop into late-stage larvae and pelagic juveniles that must then find suitable habitat for settlement and recruitment into the adult population^1^.

The pelagic phase of reef associated fishes represents an ecologically distinct phase in their life cycle, that presents unique demands on their swimming abilities. Survival during this phase is usually very low and can be dramatically altered by small changes in feeding success and predator avoidance^2^. The swimming capabilities of fishes during this early life-history stage are key to both feeding and predation aspects of their basic ecology. Furthermore, swimming capabilities determine the degree to which the individuals may influence their dispersal, and may be fundamental to their success at finding and recruiting to suitable settlement habitat.

Over the last two decades there have been a range of studies on the swimming capacities of the pelagic stages of tropical marine fishes, both during development^3–6^ and at the settlement stage^3,7–12^. Much of this work has been carried out by the authors of this data publication. A wide range of methods are available for measuring the swimming capacity of fishes, which have been reviewed extensively elsewhere (e.g., see^13–16^). Of the methods put forward, U-crit speed^17^ has been the most widely used, and represents a robust, relatively quick and repeatable measure of swimming performance. While U-crit swimming speeds are not always considered to have direct ecological relevance, they do provide a measure of relative swimming speed^13^ that can serve as a surrogate for other measures of swimming performance, such as sustained swimming speeds^18^, undisturbed swimming activity^19^, and *in-situ* speeds^16,20^. As the importance to dispersal of behaviour – in particular, swimming ability – becomes more widely recognized, researchers attempting to model dispersal require data on swimming ability of fish larvae and settlement stage fishes to parameterize their models^21^. The data presented here will be particularly useful for this purpose.

Here we publish the data that underpin much of the U-crit based publications on tropical larval and settlement stage fish swimming speeds. This includes two datasets:

1. A U-crit swimming speed dataset for settlement stage fishes, captured typically with light traps or crest nets, with a set of morphological measurements of many individuals that were swum. In some cases, the original images on which measurements were made are also available. These are the data that appear in the publications^3,8,9,11,22–25^:
2. U-crit swimming speed datasets for part of, or the entire larval development phase, based on reared larvae. For developmental data collected in Australia, larvae that were swum in experiments are not the same individuals as those for which morphological measurements were obtained, because a random sample was taken from each batch for these two different purposes. In this case morphology can only be linked to the swimming speed measurement at the batch_id level. These are the data that appear in the publications^3,4^. For data collected in Taiwan and France^6,26–28^ Ucrit and length measurements are taken from the same individuals.

In both datasets, we include a small number of previously unpublished U-crit measures to make available data, albeit limited, on additional species. Subsets of these data have also been used in a range of other comparative studies and reviews, including^7,13,15,16,18–20,28^. By making these data available in their most raw form, we hope they may prove useful for further studies of larval swimming performance, such as analyses relating to variability (including the implications for survivorship and dispersal) and other broader syntheses.

## Methods

### Settlement stage fishes

#### Specimen collection

Data for settlement stage tropical larval fishes includes 1372 swimming speed measurements, collected from >75 unique taxa across 35 families of fishes, most of which are coral reef associated as adults. The data are collected from five locations, including: South Caicos Island (Turks and Caicos Islands, Caribbean - TCI), Green Island or Magnetic Island (exact location not recorded for individual samples, Great Barrier Reef, Australia GI/MI), Lizard Island (Great Barrier Reef, Australia - LI), Calabash Caye (Turneffe Islands Atoll, Belize - BLZ), and Moorea, Society Islands (MOR) (Table 1).

**Table 1:** Sampling locations for settlement stage Ucrit data. Included are the Region, year of collection, location and associated name, as well as the total number of recorded measurements (count).

Region	Year	Location	Name	Count
Caribbean	2003	BLZ	Calabash Caye, Belize	58
Caribbean	2004	BLZ	Calabash Caye, Belize	16
Caribbean	2005	BLZ	Calabash Caye, Belize	327
Caribbean	2003	TCI	South Caicos, Turks and Caicos Island	109
GBR	1992	GI/MI	Green Island or Magnetic Island	144
GBR	2000	LI	Lizard Island, Australia	118
GBR	2001	LI	Lizard Island, Australia	217
GBR	2003	LI	Lizard Island, Australia	193
GBR	2004	LI	Lizard Island, Australia	6
GBR	2005	LI	Lizard Island, Australia	31
MOR	2010	SI	Moorea, Society Islands	152

Data from LI and TCI were obtained almost exclusively from specimens collected using light traps, placed 100–500 m meters off the leeward side of the Island, near either the School for Field Studies facilities (TCI) or the Lizard Island Research Station (LI). An additional eight specimens of newly hatched *Acanthochromis polyacanthus*, a pomacentrid species which does not have a pelagic phase, were captured with nets on the Lizard Island reefs. Data from GI/MI were obtained using a combination of light traps, beach seines, fence and dip nets.

For data collected in Moorea (Mor), specimens arriving over night from the open ocean and attempting to settle on the reef were captured in nets placed on the reef crest. In Belize (BLZ) specimens were collected using a variety of techniques including crest nets, channel nets, light traps and night-light lift nets, although most individuals were collected using light traps and crest nets. Crest net locations were those reported in^29^. Unless stated otherwise in the “notes” field of the “ucrit_sett_dat” data table (see Online-only Table 1), all U-crit measurements on individuals captured by light traps or crest nets were made on the morning of capture, usually within 6 or 12 hours (please refer to the original publications for methodological details). In a few cases, some individuals were kept in the laboratory for up to 2 days to study changes in swimming speed associated with settlement (see^24,30^), and here the post-settlement status of the larvae was recorded in the field “stage” in the “fish_id_dat” data table (see Online-only Table 1). Some specimens of the pomacentrid, *Abudefduf saxatilis* were collected with hand nets from a fish attracting device deployed over a seagrass bed from a dock and are best considered as early post-settlement individuals, although the time since settlement is unknown. All specimens of the labrid, *Clepticus parrae* were collected with hand-nets from deep fore-reefs. Although they had settled to the fore-reef an unknown period of time before capture, these individuals had yet to undergo complete metamorphosis. Data from such post-settlement individuals should be used with caution, as it is known that swimming performance in some species decreases markedly upon settlement^11,24^.

Most data were collected during the summer months (May through September for TCI and BLZ, November through February for LI), but in MOR, the data came from winter (August). In Belize, data were collected in 2003, 2004 and 2005, totalling 401 U-crit measurements. Data from TCI were collected in 2003, from 109 individuals. Data from LI represented just over half of the settlement stage swimming data (556 measurements), and were collected in 2001, 2002, 2003, 2004 and 2005. A total of 144 measurement were available from GI/MI and were collected in 1992. The 152 U-crit measurements from MOR were from 2010.

Captured settlement stage larvae/pelagic juveniles were held in fresh seawater for a minimum of 1–2 hours to reduce stress prior to swimming trials, either with an aeration stone in 24 L buckets (BLZ), or in flow-through seawater aquarium facilities at the Lizard Island Research Station (LI) and the Department of Environment and Coastal Resources at South Caicos (TCI).

#### U-crit protocol

All swimming experiments were conducted at ambient seawater temperatures, which ranged between 25 °C and 30 °C, depending on location and date.

**Fig. 1:**
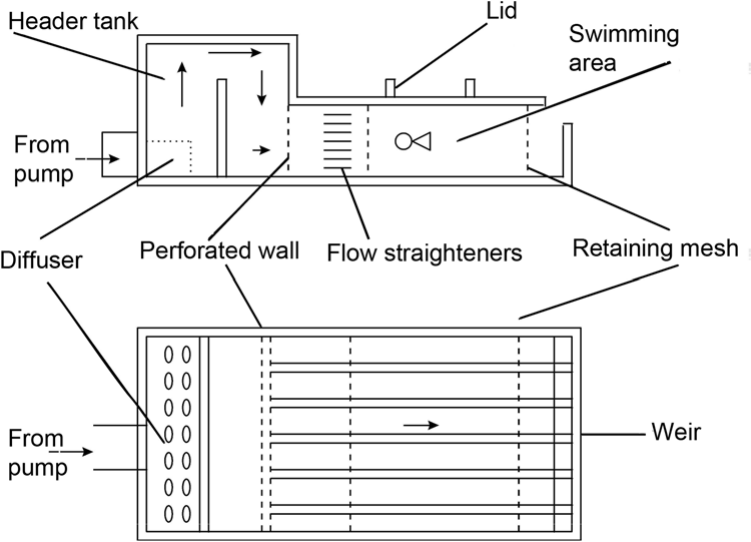
Design of the swimming channel used for settlement stage larvae, pelagic juveniles and settled juveniles. Shown are Side view (**a**) & Top view (**b**, modified from^12^). This swimming channel can be operated at up to 50 cm s^−1^. Smaller designs with three channels, or only 1 channel that could obtain higher speeds were used for swimming faster individuals. For data collected by Leis and colleagues, higher speeds were obtained by blocking some lanes using Perspex.

Settlement-stage individuals were swum using one of several swimming flumes, including a single-lane swimming chamber^11,22^, a six-lane swimming chamber^12^ (see Fig. 1), or a three-lane swimming chamber modified from the design of^12^. All swimming chambers were constructed from transparent Plexiglass (internal dimensions of swimming area: 185 mm × 50 mm × 50 mm). A removable lid, sealed with an O-ring was used to introduce fish to, and remove them from the chambers. One section of flow straighteners, 45-mm long, was placed just after the inflow in order to reduce turbulence within the chamber. Fish were retained within the swimming area by two 4.0 mm mesh metal retaining fences, which were covered with a finer mesh when required for very small larvae.

**Fig. 2:**
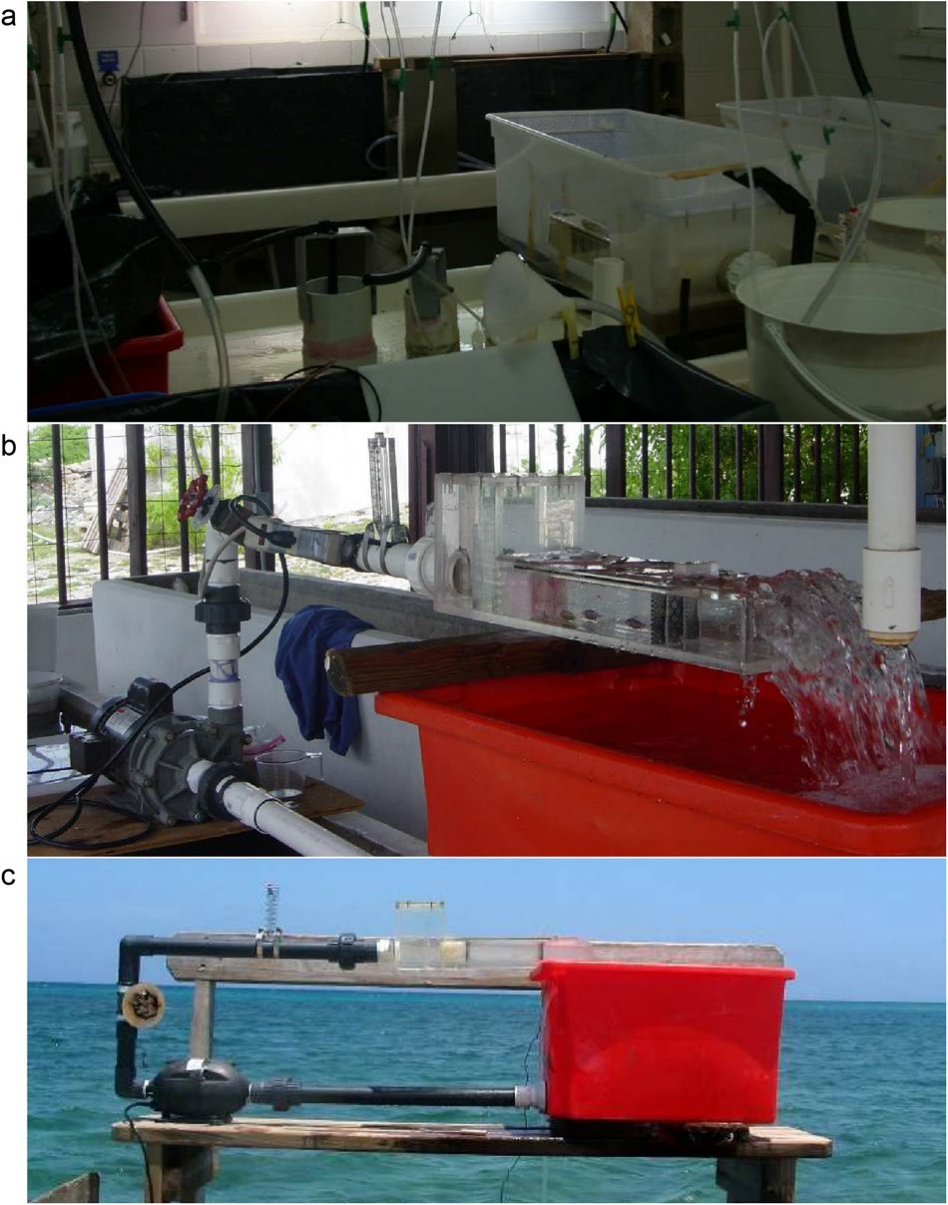
Examples of the ‘fast’ swimming flume setup at various field locations. Shown are the Lizard Island Research Station (Great Barrier Reef, Australia, **a**), the Department of Environment and Coastal Resources aquarium facilities at South Caicos (Turks and Caicos Islands, **b**), and the dock at the University of Belize Institute of Marine Field Studies at Calabash Caye (Belize, **c**).

Flow was generated using a 2.4 Kw swimming pool pump (although the size of the pump varied across the studies), or plumbed into a laboratory seawater system. The speed was set using a protractor mounted on a gate valve and calibrated using the procedures described under the technical validation section below. Faster speeds were also calibrated using an inline blue-white F-300 series flow meter. Flumes were plumbed using union valves so they could be dismantled and easily relocated and installed in field locations. Because the pumps used to run the flumes can heat the water temperature over time, they were plumbed with a minimum reservoir volume of 70 L, with a constant flow through of fresh seawater. A mercury thermometer located in the reservoir was used to ensure temperature remained ambient during the swimming trials. Example field deployments of the swimming channels at various locations can be seen in Fig. 2.

U-crit was measured by placing specimens in the swimming flume and incrementally increasing water speed until the individual could no longer maintain position for the full-time increment interval. The exact experimental protocols differed slightly among the studies. For fish measured at Lizard Island in November 2000–January 2001, November–December 2001 and South Caicos Island, and most fish at Calabash Caye, the experimental protocol followed^7^, with speed increments equivalent to approximately three total standard body lengths per second (bls^−1^) with a time interval of 2 min. This protocol was adopted because settlement stage larvae can vary substantially in size and subsequently their swimming capacity, as swimming speeds are strongly controlled by body size^4^. Aligning the speed increments with the approximate size category of fishes ensured that the overall duration of the U-crit experiment was relatively similar. For fish measured at Lizard Island during December 2003, speed increments used were 1.6 cm s^−1^ with a time interval of 5 min. At Lizard Island in 2005, specimens of *Amblyglyphidon curacao* were subjected to speed increments of 4.2 cm s^−1^ at intervals of 5 minutes. In Moorea in 2010, all individuals were subjected to speed increments of 6.1 cm s^−1^ at intervals of 2 minutes. For fish measured at Green Island and Magnetic Island speed increments used were 5 cm s^−1^ with a time interval of 5 min. At Calabash Caye an experiment was conducted to examine the impact of time increments on U-crit measurements, and the experimental protocol was recorded in this instance.

U-crit swimming speed was calculated following^17^:

1 $$U \mbox{-} crit=U+\left(t/ti\,\ast \,Ui\right)$$

Where:*U* = penultimate speed (speed increment for which the fish swam for the entire duration of the set time interval). *Ui* = the velocity increment (varied by the specific study).*t* = the time swum in the final velocity incrementti = the set time interval for each velocity increment (varied by the specific study).

While the speed increments used varied across studies in this collated dataset, previous studies have found no effect of varying the length of the time interval (ti) in terms of the resulting swimming speed between fish swum at two minute intervals and those swum at 15 minute intervals for six reef fish species^10^.

#### Sample handling and morphological measurements

After each trial specimens were anaesthetised in chilled water or using clove oil (depending on the location and according to the relevant ethics approvals) and some were photographed while still fresh to maintain body flexibility and to avoid issues with shrinkage due to dehydration associated with preserved samples. Following photographing, the samples were preserved in either 70% ethanol, 95% ethanol, or 10% buffered formalin.

**Fig. 3:**
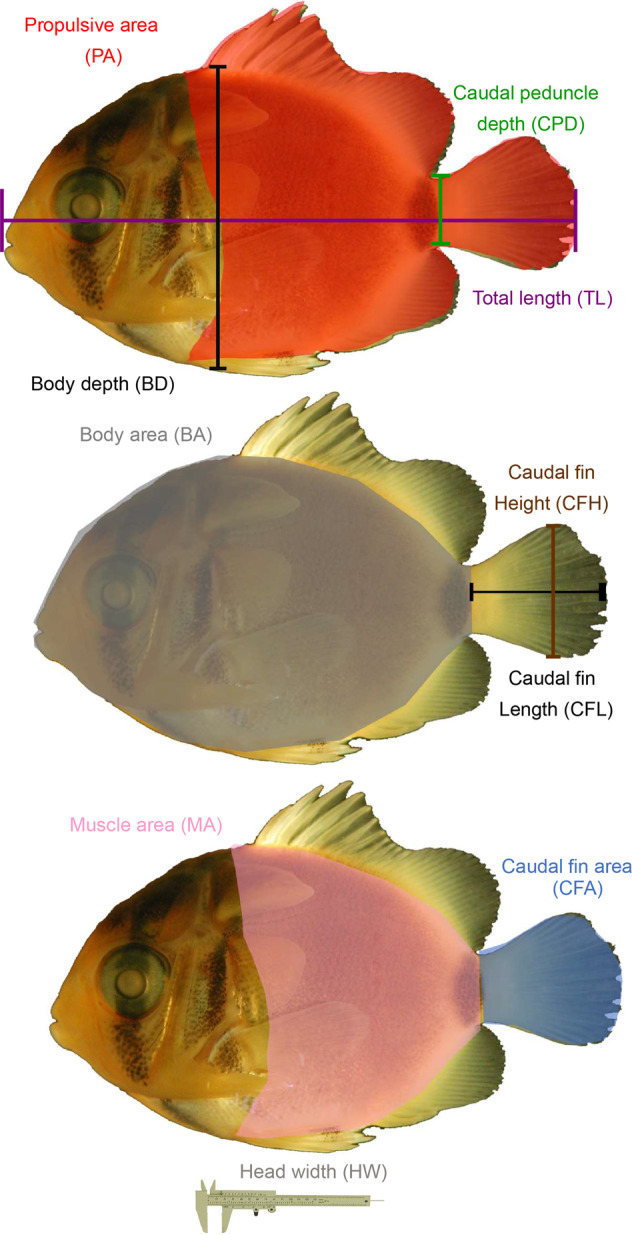
Morphological measurements of settlement stage fishes. Measurements include: total length (TL; outer edge of the caudal fin to the tip of the upper jaw), caudal fin length (CFL; tip of the caudal fin to the caudal peduncle), body depth (BD; height at the deepest region), body area (BA; area in lateral view excluding the fins), propulsive area (PA; area including the fins (naturally fully extended) but excluding the head and gut region where they are inflexible or lack overlaying muscle and cannot be used for propulsion), muscle area (MA; area excluding the fins and the head and gut region), caudal fin depth (CFD; widest section when fully extended), caudal peduncle depth (CPD; height at the narrowest point between the caudal fin and the fish’s body) and caudal fin area (CFA; area with the caudal fins naturally fully extended). Callipers were used to measure head width. Adapted from^8^.

From digital images the ImageTool (UTHSCSA 2002) software was used for image analysis. Measurements made from digital images (where available) are shown in Fig. 3, and included: total length (from the outer edge of the caudal fin to the tip of the upper jaw), caudal fin length (from the tip of the caudal fin to the caudal peduncle), body depth (the vertical height of the fish measured at the deepest region), body area (the area of the fish in lateral view excluding the fins but including the head and gut region), propulsive area (the area of the fish including the fins but excluding the head and gut region), muscle area (the area of the fish excluding the fins and the head and gut region), caudal fin depth, caudal peduncle depth and caudal fin area. All measurements were taken to the nearest 0.1 mm. Body width (at the widest region, usually the head) was also measured to the nearest 0.1 mm using vernier callipers. In some cases total lengths (TL) were measured pre-trial using callipers (BLZ, 2003 and 2004). Body length (BL, which is equivalent to SL for postflexion stages) was measured using an ocular micrometer on a dissecting microscope in some studies^23^.

### Larval development dataset (Australia)

#### Rearing protocol

Data gathered using a combination of the ‘fast’ and ‘slow’ swimming chambers (see below) on swimming abilities throughout development are available for six species, including two damselfish - *Pomacentrus amboinensis* and *Pomacentrus mollucensis* (Pomacentridae; Pomacentrinae); two cardinalfish - *Ostorhinchus (Apogon) compressus* and *Sphaeramia nematoptera* (Apogonidae); and two anemone fish - *Amphiprion percula* and *Amphiprion melanopus* (Pomacentridae; Amphiprioninae). Note that the pomacentrids have demersal eggs, whereas the apogonids orally brood their eggs.

**Fig. 4:**
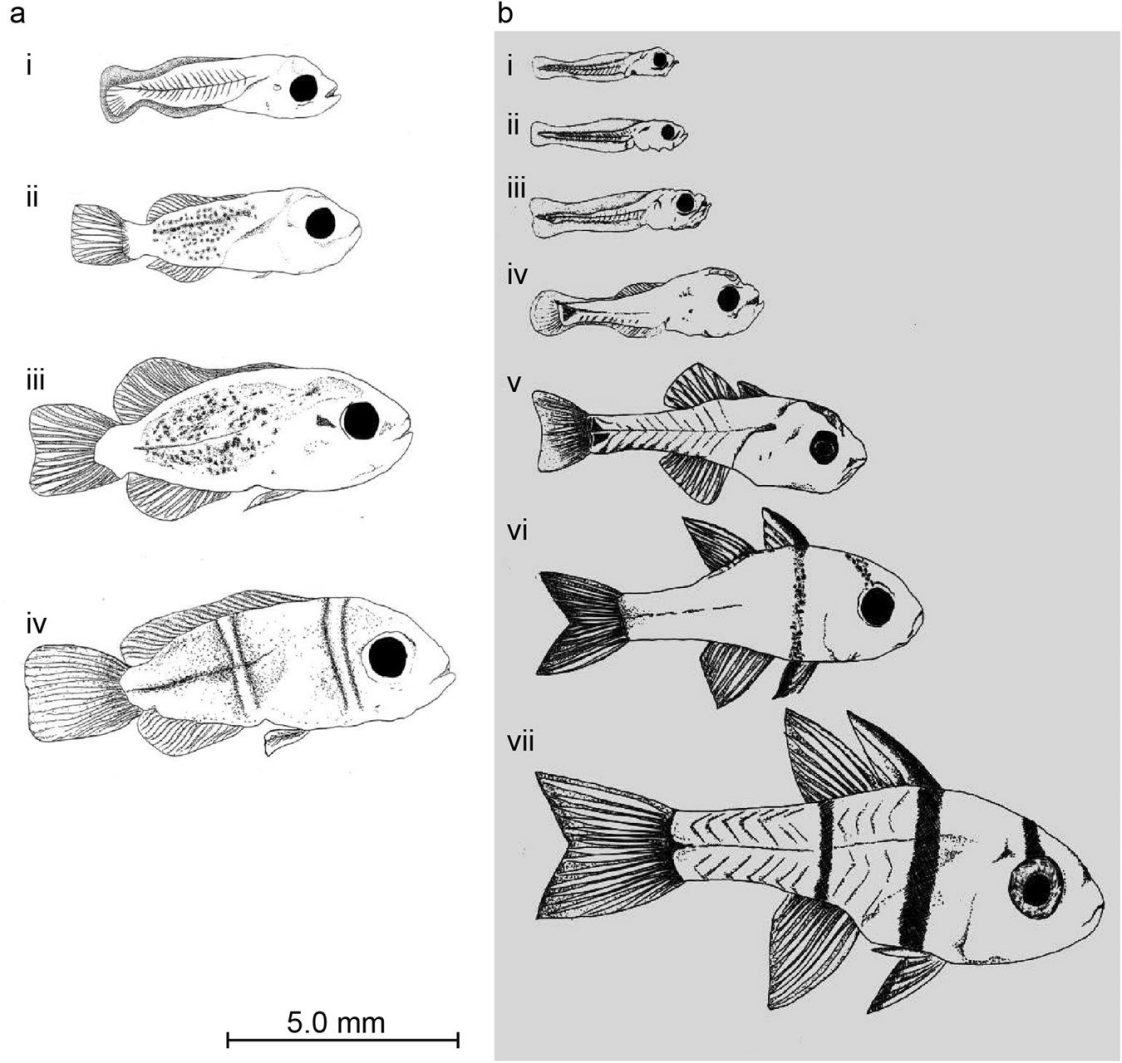
Examples of larval developmental series obtained for larvae reared at the James Cook University Aquarium facility. Showin are *Amphiprion melanopus* (**a**) and *Sphariamia nemaptopera* (**b**).

Australian specimens for assessing swimming speeds throughout larval development were obtained mostly from larvae reared at the James Cook University Aquarium facility, from adult broodstock collected from the northern Great Barrier Reef. Adult brood stock were kept in outside aquaria ranging in size from 1000 to 3000 L. The temperature of aquaria was kept between 27 and 29.5 °C, with larvae reared in the Autumn and Winter of 1998. Brood stock were fed a diet of chopped pilchards, prawns and *Ascetes* twice per day. Eggs were obtained from spawning broodstock before dark on the night of hatching and transferred to a rearing tank. Once hatched, larvae were reared and maintained in 200 L (120 × 60 × 30 cm) black painted glass aquaria that were illuminated by four “daylight” fluorescent tubes. The larvae were maintained in a 14:10 light/dark photo-period at 27.5–29 °C. Cultures of the algae *Nannochloropsis* sp. were used to green the water during the day. This kept light at the right intensity to prevent “bashing” behaviour (young larvae have a tendency to continually butt their heads against surfaces if the water is clear and the light intensity is too bright). Larvae were fed a diet of >52 micron sieved wild caught plankton, which was occasionally supplemented by rotifers and *Artemia* spp. when necessary. Larvae were fed twice per day to maintain prey densities of between 2–6 individuals per ml. Examples of ontogenetic series obtained through these rearing methods, and showing pre- and post- flexions stages are show in Fig. 4.

#### U-crit protocol

Swimming experiments for older [i.e., postflexion] larvae (see Fig. 4(a)ii–iv and Fig. 4(b)iv–vii) were carried out using the flumes described above for settlement stage fishes. However, these flumes were unsuitable for measurement of swimming capabilities of the delicate younger [i.e., preflexion] larvae (see Fig. 4(a)i and Fig. 4(b)ii,iii). Several characteristics had to be addressed in order to design equipment suitable for the measurement of the swimming capabilities of very young larvae. These included:

- The apparatus needed to produce slow flow rates while maintaining laminar flow and minimal boundary layer effects. This is because newly hatched larvae are small enough to effectively utilise the boundary layer, which is broader for slower moving water.
- The apparatus had to provide an environment suitable for very young larvae as the trauma of transferring larvae between containers can be fatal. Accordingly, stress associated with sudden changes in light intensity or water quality was minimised by “greening” the water with algae and the use of dark or clear surfaces to avoid “bashing” behaviour. In addition, the apparatus had to be set up within the immediate vicinity of the rearing tanks (or possibly in a rearing tank) to minimise the distance larvae had to be moved.

**Fig. 5:**
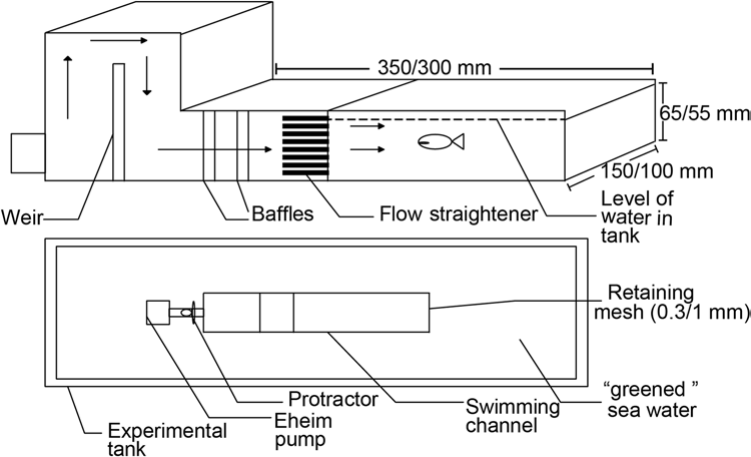
Design of swimming channels for younger larvae. Shown are side view (**a**) & Btop view (**b**). Dimensions are for the “slow” and “medium” flumes respectively.

Two swimming channels were designed and used for younger larvae that were able to meet these requirements. These were designed to operate at “slow” and “medium” speeds. Both channels were able to produce laminar flow at much slower speeds. They consisted of a much wider swimming area so that most of the water flow occurred away from the sides, maximising the area of water not influenced by boundary layer effects. Both were able to be placed in a rearing aquarium of “greened” water. This prevented the larvae from exhibiting “bashing” behaviour, minimised the distance that larvae had to be transferred and meant that there was no change in water quality between the experimental apparatus and rearing tanks (Fig. 5).

Fish were retained by a 0.3 mm mesh at the end of the swimming channel for the “slow” chamber and a 1 mm mesh for the “medium” chamber. The “slow” channel was powered by an Eheim 2,000 L per hour pump and the “medium” channel was powered by two such Eheim pumps. The speed for both channels was set using a protractor mounted on a gate valve as for the “fast” swimming chamber used for older larvae, and calibrated according to the description below under technical validation.

Each clutch of eggs from each species was raised from hatching through to settlement and experiments were performed periodically throughout this larval period, with sampling days depending on the species. The first swimming trial was conducted on day 1, approximately 12 hours after hatching. Three clutches of each species were used for each swimming trial for the species *Pomacentrus amboinensis, Sphaeramia nematoptera* and *Amphiprion melanopus* to ensure that any clutch effects were considered^4^. While multiple broodstock were available for each species, no record was made at the time from which broodstock the replicate clutches were obtained. For other species only a single clutch was available. In some cases these data included light trap caught specimens to supplement the latest settlement stage. At each experimental age for each clutch 8–12 fish were used in the swimming trials.

Larvae were subjected to incremental increases in flow rates equivalent to approximately 3 body lengths (BL) every two minutes until they could no longer maintain position, as for the experimental protocol described above for settlement stage fishes and U-crit calculated as per Eq. 1. Aligning the speed increments with the approximate size category of fishes ensured that the overall duration of the U-crit experiment was relatively similar throughout ontogeny.

#### Sample handling and morphological measurements

Fish that were swum, or siblings from the same batch at the same age, were anaesthetised in chilled water then fixed in 10% buffered formalin. After 12–48 hours, larvae were transferred to 70% alcohol and stored. Morphological measurements were carried out by capturing the image of each fish using a stereo dissecting microscope linked to a video recorder. These images were then saved as files on computer. As for settlement stage larvae, the image analysis program ImageTool was then used to measure lengths and areas for different regions of the fish.

**Fig. 6:**
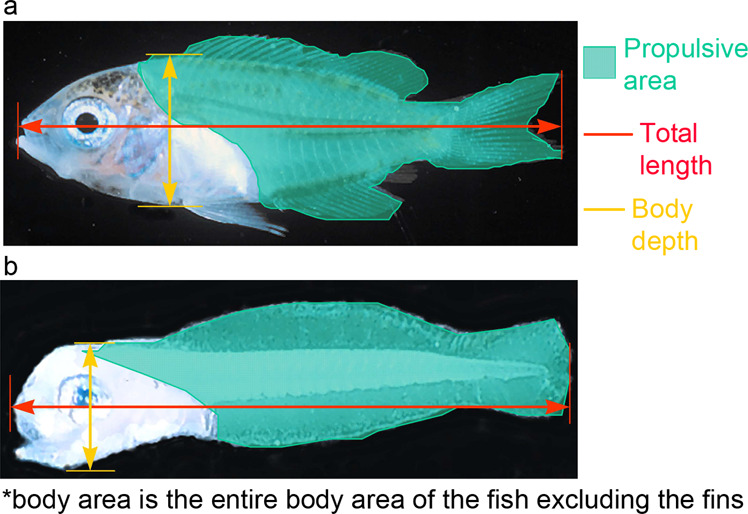
Measurements made on developmental series larvae. This includes post-flexion larvae which have developed a true caudal fin supported by a hypural plate and discrete soft rays) (**a**); and pre-flexion larvae that had no hypural plate or soft rays, but a continuous rayless fin-fold from anus to nape (**b**).

Measurements were made of total length (from the tip of the caudal fin to the tip of the upper jaw), body depth (the height of the fish measured at the deepest region), body area (the entire area of the fish excluding the fins) and total propulsive area (the area of the fish including the fins but excluding the head and gut region). The regions measured for both pre-flexion and post flexion larvae can be seen in Fig. 6.

### Larval development dataset (Taiwan and France)

Data on development of swimming in larvae of ten species of pelagic-spawning tropical species of commercially important fishes reared by aquaculturists in Taiwan^6,27,28^ and two tropical species that brood their eggs that were reared for the aquarium trade in France are included^26,28^ (see Table 2). In addition, very limited, previously unpublished data on larvae of three species of pelagic spawning, commercial species reared in Taiwan are included. The emphasis in these studies was on postflexion-stage larvae, but for some species, swimming data on preflexion and flexion-stage larvae are included. The ‘standard’ six-lane swimming chamber was used for these measurements of U-crit. For larger larvae of some species, half of the lanes were closed off to achieve the faster speeds that these larvae can achieve. Despite this adjustment, some individuals were able to swim faster than the fastest speeds the swim chamber could achieve. In these cases, the speeds are reported in the database as greater than the maximum chamber speed.

**Table 2:** Species whose U-crit swimming ontogeny were studied using reared larvae in Taiwan and France. Included are the location, family, species, the number of specimens assayed (N), the size range of the specimens, and the associated publication of the original data (where available).

Location	Family	Species	N	Size range (mm)	Publication
Taiwan	Carangidae	*Caranx ignobilis*	54	8.5–16.4	27
Taiwan	Carangidae	*Trachinotus blochii*	21	4.0–6.0	6
Taiwan	Chanidae	*Chanos chanos*	46	6.0–13.0	6
Taiwan	Ephippidae	*Platax teira*	43	4.8–10.0	6
Taiwan	Haemulidae	*Parapristipoma trilineatum*	6	23.5–28.0	
Taiwan	Haemulidae	*Pomadasys kaakan*	6	18.5–20.7	
Taiwan	Leiognathidae	*Leiognathus equulus*	72	4.5–15.5	6
Taiwan	Lutjanidae	*Lutjanus malabaricus*	50	7.0–31.0	6
Taiwan	Polynemidae	*Eleutheronema tetradactylum*	72	7.0–19.0	6,28
Taiwan	Rachycentridae	*Rachycentron canadum*	6	4.0–4.5	6
Taiwan	Serranidae	*Epinephelus coioides*	49	3.5–19.0	6
Taiwan	Serranidae	*Epinephelus fuscoguttatus*	23	12.5–27.0	6
Taiwan	Serranidae	*Epinephelus malabaricus*	15	3.0–25.0	6
Lautan	Grammatidae	*Gramma loreto*	36	5.5–10.5	26
Lautan	Pseudochromidae	*Pseudochromis fridmani*	88	4.8–12.8	

In Taiwan, larvae were obtained from commercial aquaculture farms (~22.4°N, 120.6°E) SE of Kaohsiung, southern Taiwan, in May 2004 and in May and June 2005. Rearing conditions varied with species, but most were reared in outdoor concrete or earth ponds. Exceptions were *Epinephelus* spp., which were reared in indoor concrete tanks, and *Chanos chanos* and some *Eleutheronema tetradactylum*, which were reared in outdoor concrete tanks under shade cloth. In most cases, the larvae were provided with a “natural” food source (phytoplankton and zooplankton that were resident in the pond). The aquaculturists did not maintain breeding stock, but obtained the pelagic eggs for rearing from elsewhere. The larvae obtained from the aquaculturists were placed in oxygenated plastic bags placed in insulated boxes and transported about 1 h by road to the National Museum of Marine Biology and Aquarium (NMMBA), Kenting, Taiwan (~22.1°N, 120.7°E). In the laboratory the larvae were acclimated in 40 l aquaria filled from the NMMBA seawater system. Each aquarium was fitted with an aerator and kept at ca. 25 °C. The larvae were fed twice daily with live, newly hatched brine shrimp (*Artemia* nauplii) and 50% of the total volume of water was exchanged with fresh seawater. The aquaria were cleaned daily by suctioning debris off the bottom. The species studied in Taiwan were all native to the western central Pacific, but the original brood stock may not have been obtained locally. The U-crit measurements were made in a shaded outdoor area where large tanks were located to hold adult fishes intended for either research purposes or for addition to the large public aquarium that forms part of the NMMBA campus. The extensive seawater system of NMMBA was used to supply seawater directly into the swimming chamber on a flow through basis. In some cases, this resulted in fluctuations in the calibration of the swimming chamber, which, as a result was calibrated more frequently than was normally the case. Water temperature in the chamber was recorded for each run, and all were within the range of temperatures in the nearby ocean, or in a few cases, the aquaculture ponds from which the larvae were obtained. The swimming chamber time increment interval was five minutes, with an increase in speed at each increment that varied with the flow from the seawater system and the number of lanes open in the swimming chamber, ranging from 1.6 to 5.3 cm s^−1^.

The larvae studied at Lautan Production, a small company located in Meze, France (42.4°N, 3.6°E) in September 2010 were of two species reared for the aquarium trade. *Gramma loreto* is native to the western tropical Atlantic, and the brood stock came from Cuba. *Pseudochromis fridmani* is found only in the Red Sea, but the origin of the brood stock is otherwise unknown. Both species produce ‘egg balls’ that are laid in crevices or small caves and tended by an adult until hatching. The eggs typically hatch at night with little remaining yolk and with no fin-ray development, but with mouth open and eyes pigmented. Recently hatched larvae were removed from the spawning tank to rearing tanks with constant illumination and ‘green water’ at a temperature of 26 °C to 28 °C. Cohort date is for the morning when the larvae were removed from the spawning tank. For the first 5 days rotifers were supplied, and from 6 days after hatch (DAH), the larvae were fed with *Artemia* nauplii all by Lautan employees. Temperatures in the swimming chamber were similar to those in the rearing tank. Larvae of about 5 mm to settlement size (10–12 mm) were used to measure U-crit. The swimming chamber time increment interval was two minutes, with an increase in speed at each increment of 3.2 cm s^−1^.

Larvae from Taiwan were either preserved in 75% ethanol or in some cases in Bouins Solution for future histology research. Larvae from Lautan were preserved in 75% ethanol. Measurements were made within 24–48 hours after preservation. Body length (BL) was measured on all larvae using a dissecting microscope ocular micrometer: this is Notochord Length (tip of snout to tip of notochord) for preflexion and flexion-stage larvae, and Standard Length for postflexion larvae (tip of snout to end of hypural plate). For some larvae from Taiwan additional measurements were made using Scion Image for Windows (Beta 4.02, Scion Corporation, Frederick, MD): Total Length (tip of snout to tip of posterior-most fin), Total Lateral Area (including fins) and Propulsive Area (Fig. 6), the last as defined by﻿^4^ (see Fig. 6).

### Ethic declarations

Data collated here are from a large array of studies collected across a range of institutions and locations, and to our knowledge in all cases complied with the required ethics procedures at the relevant institution at the time of data collection. Portions of this work were carried out under Australian Museum Animal Care and Ethics Approval 01/01 (JML) and James Cook Ethics Approvals A202, 402 (RF). In France, research was carried out under permits issued by CNRS to the USR 3278 CNRS/EPHE team to conduct research experiments in the field and laboratory at all locations (under the “Hygiène et Sécurité” section). In Moorea, the research was carried out under permits issued by le Délégué Régional à la recherche et à la technologie de la Polynesie française.

## Data Records

The dataset is freely available at https://github.com/open-AIMS/tropical_ucrit_data and is also lodged on figshare at 10.6084/m9.figshare.c.5486121.v2 (see^31^). While the original data were collated in MS Access as a relational database, for platform generality the dataset has been exported as seven individual*.csv* files, with irrelevant and other calculated fields removed (those only to specific historic studies).

**Fig. 7:**
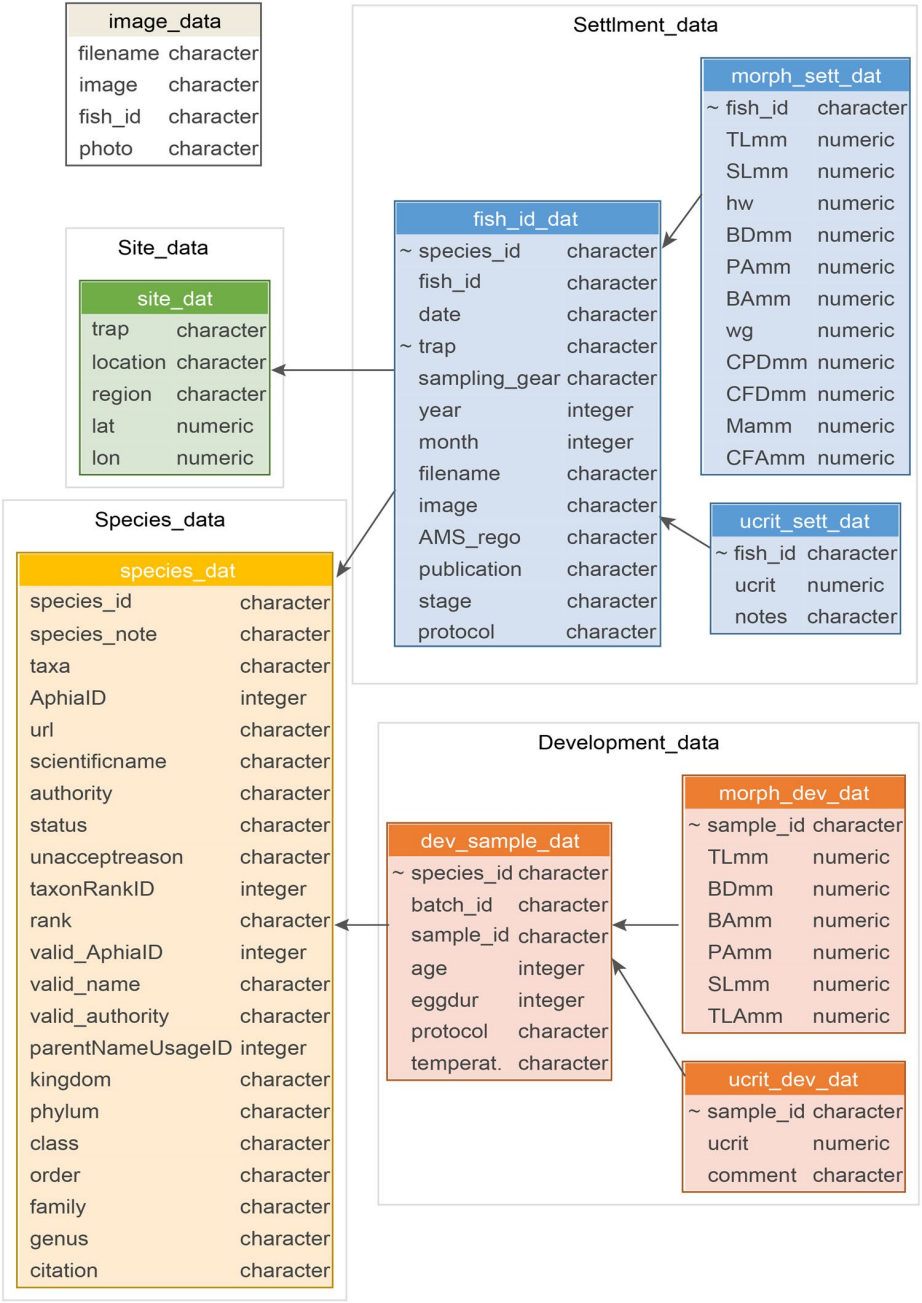
Data structure showing the individual tables and their linkages. Primary keys are underlined, and links to child tables are indicated using ~. Constructed using the datamodelr package in R (https://github.com/bergant/datamodelr).

The data comprise two higher level tables, site_data and species_data that contain location and taxon identification information respectively (Fig. 7). The settlement data consist of three tables, a higher-level fish_id_dat table with information regarding each fish measured, a ucrit_sett_dat table which contains the swimming speed data, and a morph_sett_dat table which contains the morphology data (where available) for a given fish_id (Fig. 7). The developmental data consists of three tables, a higher-level dev_sample_dat table containing the information of a given sample_id (a particular species, batch and age combination for the data collected by Fisher and colleagues in Australia, or individual fish identifier for data collected by Leis and colleagues in Taiwan and France), a ucrit_dev_dat table containing the swimming speed data, and a morph_dev_dat table which contains the morphology data (where available) for a given sample_id (Fig. 7). There is a table of image_data, and where matches were possible these can be linked to the U-crit data through the fish_id field. A description of all fields can be found in the meta-data reported in Online-only Table 1. Source data are also included, as well as the R scripts used to process and describe these data (check_data, document_data). These are included purely for repeatability/transparency purposes for interested users, however our intent is for most users to simply use the files described in Fig. 7 and Online-only Table 1 that are contained in the clean_data folder.

## Technical Validation

### Swimming chamber calibration

#### Settlement stage swimming channels

**Fig. 8:**
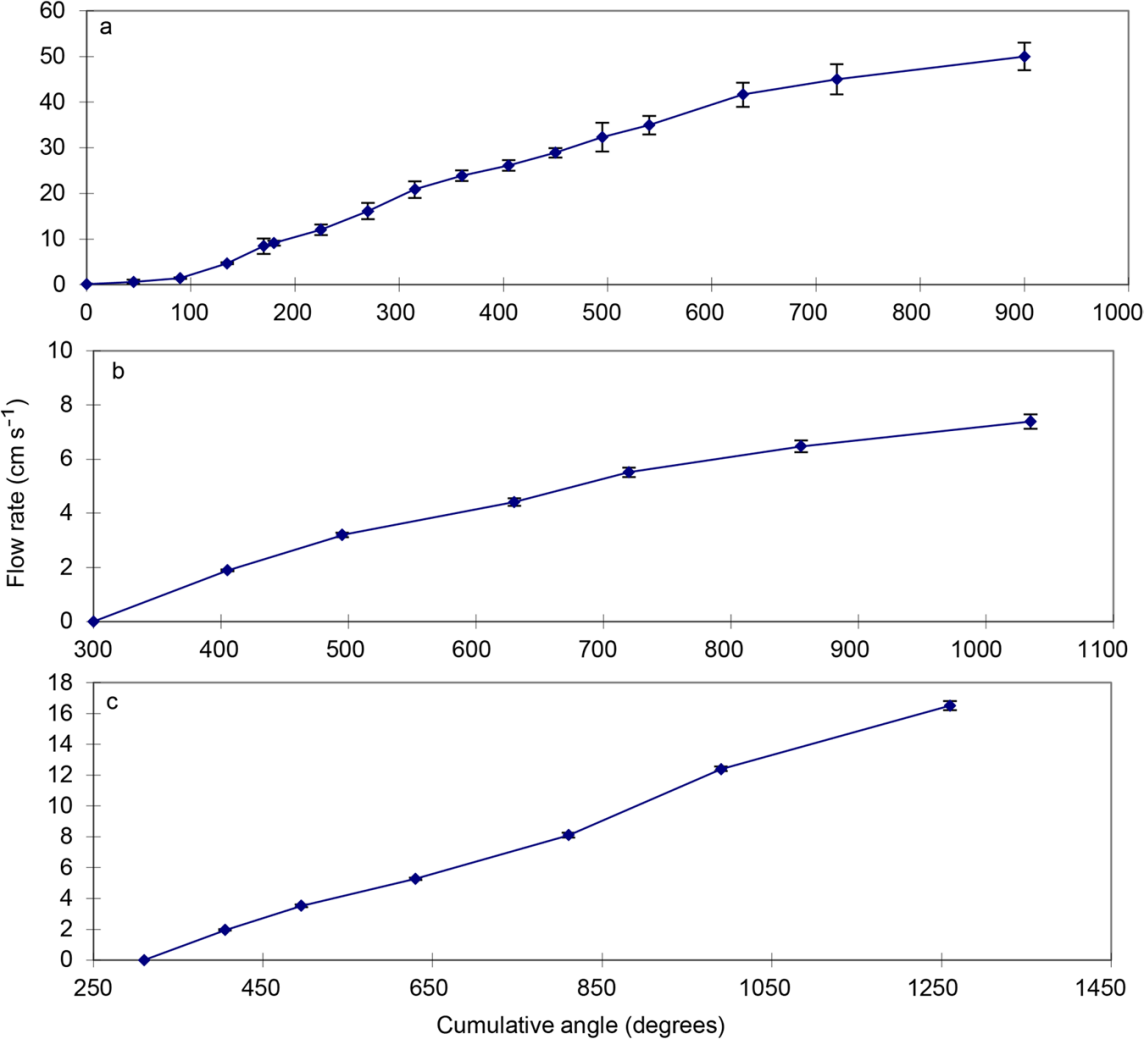
Initial working calibrations for the swimming flumes. This includes the fast (**a**, settlement stage fishes, Fig. 1), slow (**b**) and medium (**c**) swimming channels for younger larvae (Fig. 5). Date are mean with SE for n = 3 replicates and were collected during the work reported in^4^.

**Fig. 9:**
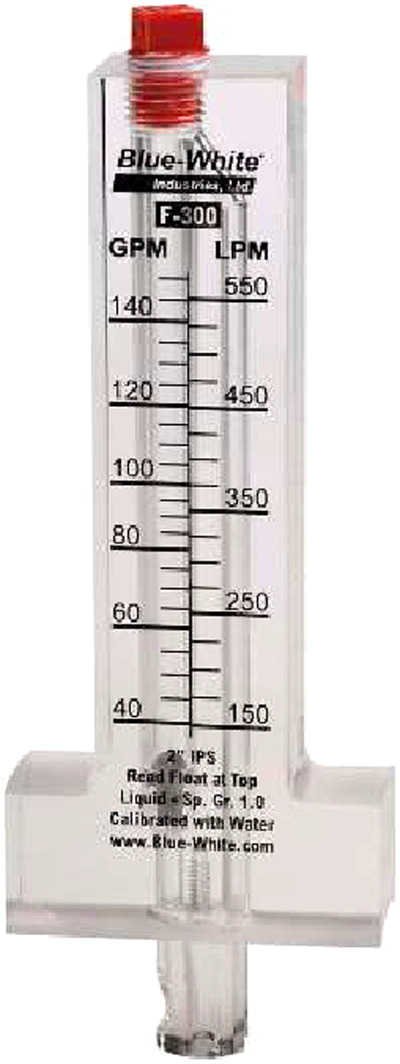
F-300 series pipemount flow meter used to aid calibration of the faster swimming channels at higher speeds.

For the chambers used for older and settlement stage fishes (see Fig. 4(a)i–iv and Fig. 4(b)i–vii) calibration of the water speed through the chamber was carried out by recording the volume of water passing through the chamber over a set period of time for different angles on the protractor. This was achieved using a stop-watch and bucket or similar. The estimated flow rate was divided by the sum of the cross-sectional area of each channel to calculate speed. In initial studies^4^ this calibration was performed five times and ANCOVA indicated that there was no significant difference between the calibrations (F_4,92_ = 4.7, P = 0.25), and the final calibration graph produced was based on the mean of all trials (see Fig. 8a). Further calibrations of this and the other settlement stage swimming chambers were generally carried out using three replicate measurements at each speed. The swimming speeds of late-stage reef fishes of some families are very fast. As the bucket and stop-watch method of calibration becomes highly variable at very fast water flow, for field-based studies of these later stage fishes^3,9,10,18^ calibrations were based on an F-300 series pipemount flow meter (Fig. 9) at faster speeds. Calibrations were carried out whenever the equipment was set up for each separate field campaign, resulting in many calibration graphs which are not all shown here.

Note that some individuals of five species could swim faster than the maximum speed of the swimming flume used at Lizard Island in 2003/2004. In such cases, we used the maximum speed obtained as the nominal U-crit value for the individual, although a note that this was the maximum speed of the flume was recorded. In Moorea and Taiwan, the speed of fishes that could swim faster than the maximum speed of the flume is given as >X cm s^−1^ (X being the maximum flume speed).

#### Early larval stage swimming channels

Calibration of the two swimming channels for younger larvae (see Fig. 4(a)i and Fig. 4(b)i–iii) was carried out by videoing neutral density particles (un-expanded polystyrene balls) travelling through the swimming channel at different settings of the gate value. This video was analysed frame by frame to determine the distance covered by particles between frames. Multiple recordings for each angle were conducted and there was found to be no significant difference in the speed of each trial (F2,244 = 2.14, P = 0.08 and F2,465 = 0.3, P = 0.55 for the “slow” and “medium” swimming channels respectively and data were pooled for each calibration accordingly, Fig. 8b,c).

#### Boundary layer characterisation

The boundary layer size and flow characteristics of the six channel swimming chamber used for settlement stage fishes (see Fig. 1) was previously determined by^12^ and were not repeated for the additional chambers that were based directly on that design.

**Fig. 10:**
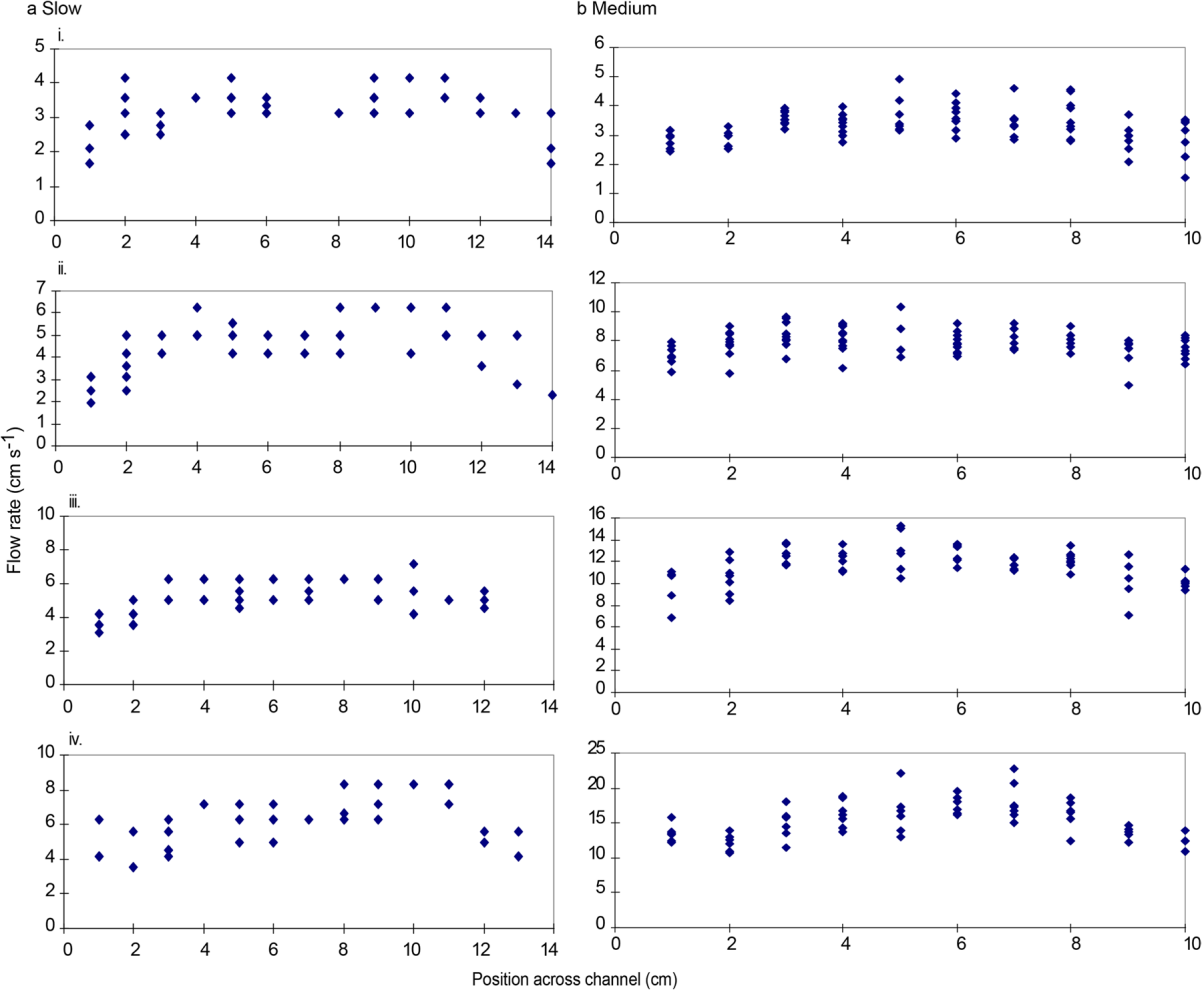
Flow rates at different positions across the swimming channels. Shown are speeds for the “slow” (**a**) and “medium” (**b**) swimming flumes for early stage larvae (see Fig. 4(a)i and Fig. 4(b)i-iii) at slow (i) through to the fastest (iv) speeds. These calibrations were carried out during the work reported in^4^.

For the two new swimming chambers developed for use with younger larvae, the speed of particles travelling at every 1 cm across the swimming channel, as well as from the top to the bottom of the channel was recorded and plotted to investigate possible asymmetry and boundary effects in the swimming channel at different speeds. For both the “slow” and “medium” swimming channels it was found that there was no significant differences in the flow rate at four different heights above the bottom of the swimming channel (F_3,95_ = 1.3, P = 0.3 and F_3,108_ = 0.3, P = 0.82 for the “slow” and “medium” swimming channels respectively, see Fig. 10). For both swimming channels it was found that there was a noticeable boundary effect near the sides (F_13,254_ = 10.95, P = 0.05 and F_9,465_ = 17.3, P < 0.0001 for the “slow” and “medium” swimming channels respectively, see Fig. 10). An effect was detected within 3 cm of the sides for the “slow” swimming channel and 2 cm from the sides for the “medium” swimming channel. Consequently, fish were censused throughout the experiment to determine their locations and only those swimming for at least 90% of the time within the region outside the boundary layer were included in the experiments/data. In only six cases were larvae seen to use the boundary layer. The calibration graphs used (see Fig. 8b,c) were based on measurements of water speed for all centimeters across the chamber within 3 cm of the sides for the “slow” swimming channel and within 2 cm of the sides for the “medium” swimming channel. Larvae were encouraged to remain within the centre of the channel by differential illumination to avoid the boundary layers.

### Larval behavioural issues

In all experiments, specimens were transferred to the appropriate swimming chamber and allowed to acclimatise for 2–15 minutes at either a very low or zero speed. Fish that exhibited symptoms of stress such as erratic swimming behaviour, laying on the bottom or in corners of the channel, or clinging to the sides or surface of the swimming channel were excluded from experiments.

### Morphological measurements

#### Images

A total of 979 images of settlement stage fishes used in swimming experiments in TCI, BLZ and the LI were retrieved from various sources, 571 of which can be matched to U-crit swimming data through the fish_id field. Inconsistent labelling issues mean that not all images can be automatically paired with samples. Raw images used for morphological measurements of development stage larvae could not be located.

#### Issues with sample preservation

**Fig. 11:**
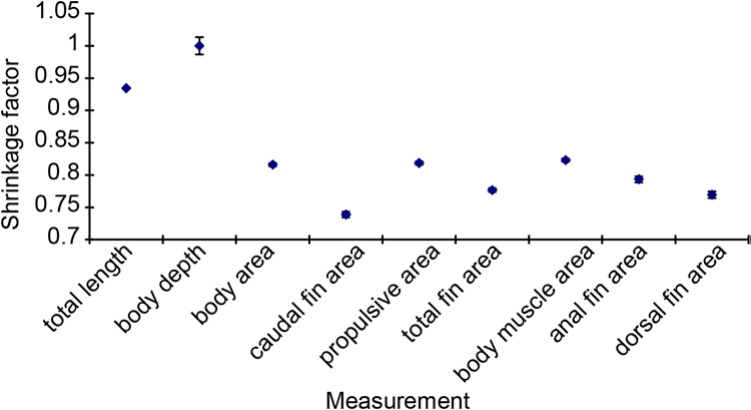
Mean shrinkage factor for each variable separately. All species of all ages have been grouped. Data collected during the work reported in^4^.

Morphological measurements of development stage larvae were carried out using preserved samples. Although larvae exhibit some degree of shrinkage (approx. 10%), this varies among ages and parameters^32^. A shrinkage factor was calculated for each experimental age of each species. This was estimated by measuring a fresh sample of larvae as soon as they were collected and then measuring the same individuals once they had been fixed in 10% buffered formalin and stored in 70% alcohol for at least 3 days. The shrinkage measurements were found to be highly variable among the different morphological measures (see Fig. 11), and there were also differences among different ages and species, making application of a shrinkage correction complex. For consistency and simplicity all values are based on raw (non-corrected) measurements, although it must be noted that for some measurements this would represent a substantial underestimate of the parameter value, particularly for measurements of fin area (Fig. 11).

#### Poor images and expert judgement

In the field it was often difficult to obtain clear images with well displayed fins for measurements, and the quality of some images was sometimes quite poor. Expert judgement was used to estimate the likely shape of fishes where portions of the fishes were missing, or fins not properly displayed.

### Species identification

Where possible, specimens were identified to species level. At Lizard Island this was achieved with the aid of^33^, comparison to voucher specimens at the Australian Museum, and in some cases by growing out specimens to juvenile stages. At South Caicos specimens were identified to species by comparison to newly settled juveniles and online identification guides. At Calabash Caye individuals were identified to the lowest taxonomic level possible by either keying out preserved individuals, identifying individuals at the time of capture based on distinct colourations, or by rearing individuals in aquaria until their juvenile colourations revealed their identity. For some labrid and apogonid larvae from Moorea^24,25^ genetic barcode ID was made, whereas eels were identified by Dr Michael Miller, University of Tokyo^34^.

Fish that could not be identified to species were identified into clearly distinguishable types and allocated a unique identification number so specimens could be grouped as similar morphological types. The lowest taxonomic resolution available was also recorded, which in most cases was at the genus level, but in some cases, this was at the family level. This data was collected some time ago and, in some cases, taxonomic classification of species and genera has changed considerably in the intervening years since initial identifications. We used a computer script to correct for any misspelling of species names, and to generate a “taxa” field that was used to find the current valid name (at the time of publication) according to the World Registry of Marine Species (WoRMS^35^) using the “worms” package in R^36^. Please see the information for the WoRMS database for meta data concerning the various fields (shown in Online-only Table 1). The field valid_name indicates the valid species name for the taxon, rank indicates the taxonomic rank of that valid name (Species, Genus or Family respectively), and taxa is the name as used in the original dataset and associated publications.

## Usage Notes

The R file *Use_data.R* shows some examples of how to read in the raw data, link the various elements, and make some basic summaries. For the settlement stage data linking data is relatively straightforward as there is a 1:1 relationship between the morphology and the U-crit datasets, which can be joined through their fish_id identifier. Note that there are multiple readings for a given sample_id for all developmental data collected in Australia for both the U-crit and morphology data, and these data cannot be matched directly at the 1:1 level (that is, individual fish are not identified in this dataset). For these data, summaries must be made at the sample_id level for either U-crit or the morphology information before they can be joined and exported.

For those readers not proficient in R we have included some ready to use tables in the github folder. This includes “sett_dat.csv”, which is a fully joined settlement stage dataset; and “dev_dat_morph.csv” and “dev_dat_ucrit.csv” which contain fully joined development datasets aggregated at the sample_id level, with the U-crit data summarized as a mean and the morphology data summarised as a mean respectively.

## Data Availability

All R code are available at https://github.com/open-AIMS/tropical_ucrit_data.

## Online-only Table

**Online-only Table 1:** Meta data for all tables and fields contained in the data set.

Table	Column	Type	Description
dev_sample_dat	species_id	character	Unique ID for a specific taxon
	batch_id	character	Unique clutch identifier.
	sample_id	character	A unique sample identifier specific to a given clutch, for a given species at a specific sample age.
	age	integer	Age of the sample in days since hatching.
	eggdur	integer	Approximate egg duration of the species
	protocol	character	NA
	temperature	character	NA
fish_id_dat	species_id	character	Unique ID for a specific taxon
	fish_id	character	Unique identifier of a particular fish sample.
	date	character	Date the sample was used in experiment.
	trap	character	Unique ID for the specific light trap, crest net or site from which the fish sample was obtained.
	sampling_gear	character	The sampling gear used to obtain the sample.
	year	integer	year the sample was used in experiment.
	month	integer	Month the sample was used in experiment.
	filename	character	Likely file name of image of sample.
	image	character	Likely image name.
	AMS_rego	character	Australian Musesum Sydney registration number
	publication	character	Most likely publication in which data appear
	stage	character	Most likely developmental stage of larvae
	protocol	character	NA
image_data	filename	character	Likely file name of image of sample.
	image	character	Likely image name.
	fish_id	character	Unique identifier of a particular fish sample.
	photo	character	NA
morph_dev_dat	sample_id	character	A unique sample identifier specific to a given clutch, for a given species at a specific sample age.
	TLmm	numeric	Total length in mm. See methods for details
	BDmm	numeric	Body depth in mm. See methods for details.
	BAmm	numeric	Body Area in mm. See methods for details.
	PAmm	numeric	Propulsive Area in mm. See methods for details
	SLmm	numeric	Standard length in mm. See methods for details
	TLAmm	numeric	NA
	fish_id	character	Unique identifier of a particular fish sample.
	TLmm	numeric	Total length in mm. See methods for details
	SLmm	numeric	Standard length in mm. See methods for details
	hw	numeric	Head width in mm, measured using calipers. See methods for details
	BDmm	numeric	Body depth in mm. See methods for details.
	PAmm	numeric	Propulsive Area in mm. See methods for details
	BAmm	numeric	Body Area in mm. See methods for details.
	wg	numeric	Sample weight in grams
	CPDmm	numeric	Caudle peduncle depth in mm. See methods for details
	CFDmm	numeric	Caudal fin depth in mm. See methods for details.
	Mamm	numeric	Muscle area in mm. See methods for details
	CFAmm	numeric	Caudal fin area in mm. See methods for details.
site_dat	trap	character	Unique ID for the specific light trap, crest net or site from which the fish sample was obtained.
	location	character	An abbreviation indicating the location where the data were collected, values includs BLZ (Belize), TCI (Turks and Caicos Islands), LI (Lizard Island), GI/MI (Green Island or Magnetic Island), SI (Society Islands) and JCU (James Cook University Research Aquarium).
	region	character	Either Caribbean or GBR (Great Barrier Reef).
	lat	numeric	approximate value of the latitude of the trap/site in decimal degrees.
	lon	numeric	approximate value of the latitude of the trap/site in decimal degrees.
species_dat	species_id	character	Unique ID for a specific taxon
	species_note	character	Notes on taxa identification
	taxa	character	The taxa name used to search for current taxonomic information in the [**WORMS database**](http://www.marinespecies.org/)
	AphiaID	integer	Field retrieved from the [**WORMS database**](http://www.marinespecies.org/) for that taxa
	url	character	Field retrieved from the [**WORMS database**](http://www.marinespecies.org/) for that taxa
	scientificname	character	Field retrieved from the [**WORMS database**](http://www.marinespecies.org/) for that taxa
	authority	character	Field retrieved from the [**WORMS database**](http://www.marinespecies.org/) for that taxa
	status	character	Field retrieved from the [**WORMS database**](http://www.marinespecies.org/) for that taxa
	unacceptreason	character	Field retrieved from the [**WORMS database**](http://www.marinespecies.org/) for that taxa
	taxonRankID	integer	Field retrieved from the [**WORMS database**](http://www.marinespecies.org/) for that taxa
	rank	character	The taxonomic rank retrieved from the [**WORMS database**](http://www.marinespecies.org/) for that taxa
	valid_AphiaID	integer	Field retrieved from the [**WORMS database**](http://www.marinespecies.org/) for that taxa
	valid_name	character	Valid taxon name for that recorded taxa. Will be same as ‘taxa’ unless that is no longer accepted according to the [**WORMS database**](http://www.marinespecies.org/)
	valid_authority	character	Field retrieved from the [**WORMS database**](http://www.marinespecies.org/) for that taxa
	parentNameUsageID	integer	Field retrieved from the [**WORMS database**](http://www.marinespecies.org/) for that taxa
	kingdom	character	The taxonomic kingdom retrieved from the [**WORMS database**](http://www.marinespecies.org/) for that taxa
	phylum	character	The taxonomic phylum retrieved from the [**WORMS database**](http://www.marinespecies.org/) for that taxa
	class	character	The taxonomic class retrieved from the [**WORMS database**](http://www.marinespecies.org/) for that taxa
	order	character	The taxonomic order retrieved from the [**WORMS database**](http://www.marinespecies.org/) for that taxa
	family	character	The taxonomic family retrieved from the [**WORMS database**](http://www.marinespecies.org/) for that taxa
	genus	character	The taxonomic geuns retrieved from the [**WORMS database**](http://www.marinespecies.org/) for that taxa
	citation	character	Field retrieved from the [**WORMS database**](http://www.marinespecies.org/) for that taxa
ucrit_dev_dat	sample_id	character	A unique sample identifier specific to a given clutch, for a given species at a specific sample age.
	ucrit	numeric	Swimming speed measurement, See methods for more details.
	comment	character	NA
	fish_id	character	Unique identifier of a particular fish sample.
	ucrit	numeric	Swimming speed measurement, See methods for more details.
	notes	character	notes taken during measurements
